# Supplementary material for: Selection of memory clinic patients for CSF biomarker assessment can be restricted to a quarter of cases by using computerized decision support, without compromising diagnostic accuracy
Source: PLoS One. 2020 Jan 15;15(1):e0226784. doi: 10.1371/journal.pone.0226784 (PMC6961870; doi:10.1371/journal.pone.0226784)
Supplement: S1 File — (PDF) [file pone.0226784.s001.pdf]

S1 File. Probability of correct class (PCC)

DSI defines the patient's similarity to each diagnostic group. For example, the following DSI values could have been measured for a patient: DSI=0.75 for AD, DSI=0.67 for FTD, DSI=0.45 for VaD and DSI=0.32 for controls. The interpretation is that AD is the most similar diagnostic group, but the question is how confident we can be about this suggestion. *Probability of correct class* (PCC) for this patient measures the probability that the diagnosis suggested is correct when the highest DSI value is 0.75 and the second highest DSI value is 0.67.

Intuitively, one could take a large database of previously diagnosed patients, search all cases with the highest DSI value close to 0.75 and the second highest close to 0.67, and calculate the share of database cases for which the diagnostic group of the highest DSI value corresponds to the ground truth diagnosis. The open question is which cases are considered to be "close" and included in defining PCC.

Formally, PCC is defined by maximizing the likelihood function of the observed data:

$$\mathcal{L}(\theta|x) = PCC_{\theta}(x)$$

where θ denotes the parameters used to estimate the probability function *PCC(x)* and *x* denotes a vector containing the highest DSI value and the difference to the second highest DSI value, {$DSI_{max,}$ ΔDSI). Maximizing likelihood defines a probability function that is the most likely for the observed data. If we consider all observed data samples independent, the likelihood of measuring all the cases is

$$PCC_{\theta}(x) = \prod_{i=1}^{n} PCC_{\theta,i}(x)$$

Maximizing this function is similar to maximizing its logarithm

$$\log PCC_{\theta}(x) = \sum_{i=1}^{n} \log PCC_{\theta,i}(x)$$

Probability density function *PCC(x)* is estimated from n d-dimensional samples $\boldsymbol{x}_i$ (observed data) using a Gaussian kernel:

$$PCC_{\boldsymbol{H}}(x) = \frac{1}{n}\sum_{i=1}^{n} K_H(\boldsymbol{x} - \boldsymbol{x}_i) \quad (1)$$

where

$$K_H(x) = (2\pi)^{-d/2}|\boldsymbol{H}|^{-1/2}e^{-\frac{1}{2}\boldsymbol{x}^T\boldsymbol{H}^{-1}\boldsymbol{x}}.$$

In this case, θ is the width of the kernel, H, and *PCC(x)* estimates the probability of the class "correctly classified". As we want to maximize balance accuracy, i.e., not to prefer the most prevalent diagnoses, each sample is weighted based on the share of all samples in the class. Log-likelihood is optimized using cross-validation: samples are divided using a random selection to a training and test sets. The probability function is estimated using the training set with a specific kernel size and likelihood is computed using the test set. This is repeated multiple times and the kernel width maximizing the sum of likelihoods over all iterations is chosen. Because the size of the database is still relatively limited, we chose to estimate a one-dimensional kernel (circle – one parameter) instead of a two-dimensional kernel (ellipse – two parameters). Figure S1 shows an example on estimating PCC for a database.

Figure S1. Example on estimating PCC.

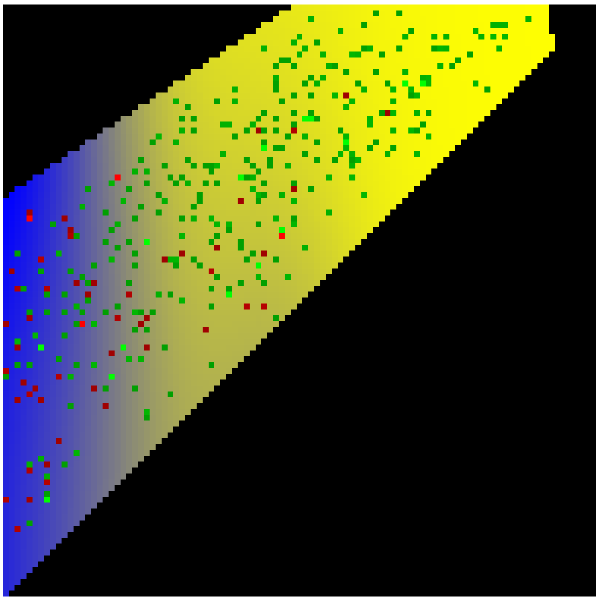

The vertical axis shows the highest DSI value (0.5-1.0) and the horizontal axis the difference between the two highest DSI values (0.0-0.67). The probabilities are color coded from blue (p=0.5) to yellow (p=1.0). The dots show all the samples: green are correctly classified cases and red ones are wrongly classified cases. The shade of the green and red dots indicates the class: the darker dots have more cases in the class (less weight in estimating the probability) than the brighter dots. The black background indicates the value pairs (DSI_{max}, ΔDSI) that are not possible. PCC shows that when DSI_{max} and/or ΔDSI are high, almost all points in the neighborhood are green and PCC starts to get close to one.
