# Supplementary figures and images for: Selection of memory clinic patients for CSF biomarker assessment can be restricted to a quarter of cases by using computerized decision support, without compromising diagnostic accuracy
Source: PLoS One. 2020 Jan 15;15(1):e0226784. doi: 10.1371/journal.pone.0226784 (PMC6961870; doi:10.1371/journal.pone.0226784)

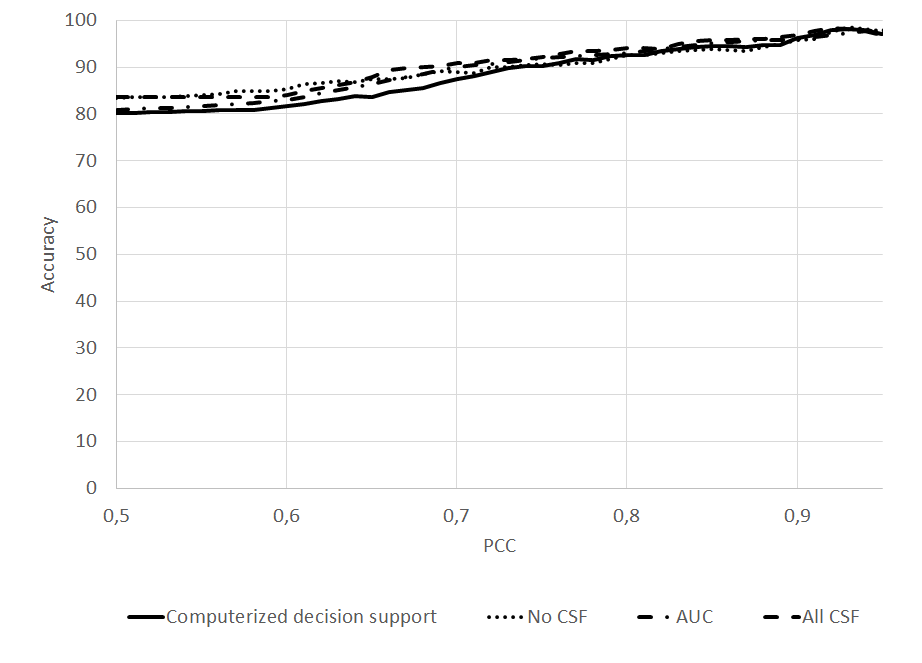

Supplement: S1 Fig — PCC: probability of correct class Solid lines show results for the computerized decision support (Fig 1A), dotted lines show results for using no CSF, but only neuropsychology, MRI and APOE (Fig 1B), dashed dotted lines show results for AUC (Fig 1C) and dashed lines using all data (Fig 1D). (TIF) [file pone.0226784.s002.tif]
